# A nomogram risk assessment model to predict the possibility of type II endoleak-related re-intervention after endovascular aneurysm repair (EVAR)

**DOI:** 10.1038/s41598-022-27356-8

**Published:** 2023-01-02

**Authors:** Zongwei Liu, Yonghui Chen, Yafei Qin, Jiaxue Bi, Jiaxin Wang, Fang Niu, Xiangchen Dai

**Affiliations:** 1 Department of Vascular Surgery, Tianjin Medical University General Hospital, Tianjin, China; 2 Department of Pharmacology, Tianjin Key Laboratory of Inflammation Biology, Key Laboratory of Immune Microenvironment and Disease (Ministry of Education), School of Basic Medical Sciences, Tianjin Medical University, Tianjin, China; 3 Department of Vascular Surgery, Henan Provincial People’s Hospital, The Affiliated People’s Hospital of Zhengzhou University, Zhengzhou, Henan China; 4 Division of Vascular Surgery Center, Sichuan Academy of Medical Sciences & Sichuan Provincial People’s Hospital, School of Medicine, University of Electronic Science and Technology of China, Chengdu, 610072 Sichuan China; 5 Present Address: Department of Vascular Surgery, Tianjin Medical University General Hospital, No. 154 Anshan Road, Heping District, Tianjin, China

**Keywords:** Aneurysm, Cardiovascular diseases

## Abstract

This study aimed to develop and validate a novel nomogram risk assessment model to predict the possibility of type II endoleak (T2EL)-related re-intervention. The data of 455 patients with abdominal aortic aneurysms who underwent elective endovascular aneurysm repair (EVAR) procedures between January 2018 and December 2021 at our single center were retrospectively reviewed. Following the implementation of exclusion criteria, 283 patients were finally included and divided into T2EL-related re-intervention (n = 42) and non-T2EL (n = 241) groups. The overall T2EL-related re-intervention rate for 283 patients was 14.8% (42/283). Using multivariate analysis, significant risk factors for re-intervention included age (OR, 1.172; 95% CI, 1.051–1.307; *P *= 0.004), smoking (OR, 13.418; 95% CI, 2.362–76.215; *P *= 0.003), diameter of inferior mesenteric artery (IMA) (OR, 21.380; 95% CI, 3.060–149.390; *P *= 0.002), and number of patent lumbar arteries (OR, 9.736; 95% CI, 3.175–29.857; *P *< 0.001). The discrimination ability of this risk-predictive model was reasonable (concordance index [C-index] = 0.921; 95% CI, 0.878–0.964). The Hosmer–Lemeshow goodness of fit test was performed on the model, and the chi-square value was 3.210 (*P *= 0.920), presenting an excellent agreement between the model-predicted and observed values. The receiver operating characteristic (ROC) curve identified that the risk thresholds of re-intervention were a diameter of > 2.77 mm for the diameter of the inferior mesenteric artery and a proportion of < 45.5% for thrombus volume in the aneurysm sac. This novel nomogram risk assessment model for predicting the possibility of patients’ T2EL-related re-interventions after EVAR should be helpful in discriminating high-risk patients. Two novel risk thresholds may imply a higher possibility of T2EL-related re-intervention after EVAR.

## Introduction

Endovascular aneurysm repair (EVAR) has been widely accepted for the treatment of abdominal aortic aneurysm (AAA) because of its lower postoperative mortality, shorter in-hospital duration, and rapid recovery compared with open surgical repair, which has been identified by prospective clinical trials including EVAR-1^1^, DREAM^2^, OVER^3^, and ACE^4^. However, the advantages of EVAR have gradually declined over time because of a series of complications. Endoleak, defined as incomplete exclusion of the aneurysmal sac from circulation, was first proposed by White^5^ in 1996 and classified as I–V^6^. Re-intervention is required for endoleaks, and type II endoleak (T2EL), defined as that arising from side branches of the excluded aneurysm, is the most common type of endoleak after EVAR, with the largest observational study and a recent meta-analysis showing an occurrence rate of T2EL between 10.2%^7^ and 29.0%^8^. However, not all T2ELs require re-intervention. According to the recent guidelines of the United States^9^ and Europe^10^, a conservative approach is appropriate for isolated T2EL without sac expansion, while intervention is recommended when sac enlargement is > 10 mm. This recommendation differs from that with pre-EVAR, during which a sac enlargement of > 5 mm over 6 months may represent a relative indication for treatment. It is difficult to predict which patients with T2EL need re-intervention after EVAR treatment based on preoperative data. Thus, the purpose of this study was to predict the risk rate of re-intervention in patients with T2EL based on the cohort at our center using a novel nomogram prediction model to provide a new strategy for T2EL management.

## Methods and materials

### Patient selection and follow-up

**Figure 1:**
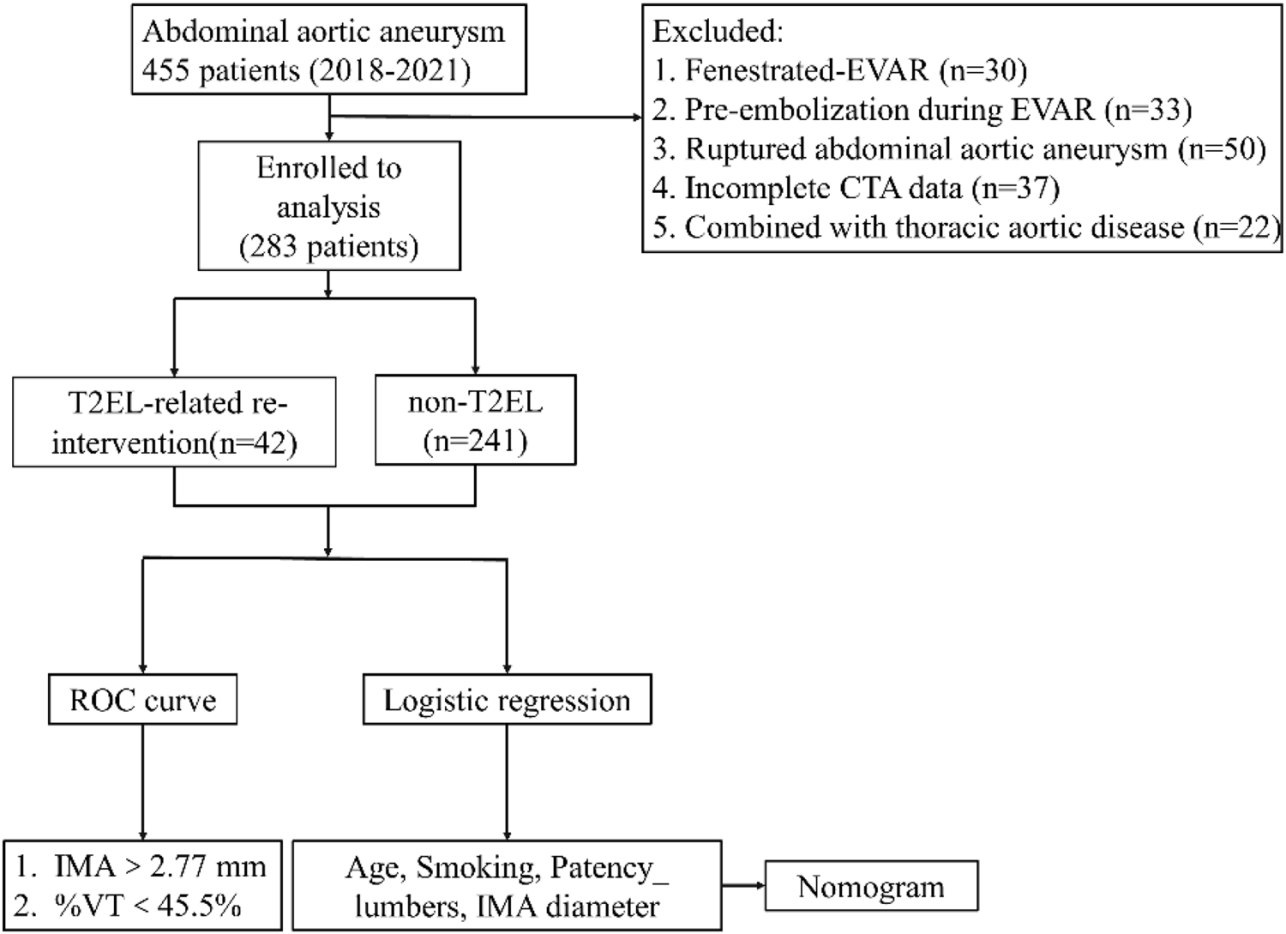
Flow chart Retrospective analysis process and patient exclusion criteria.

This retrospective study complied with the declaration of Helsinki and was approved by Tianjin Medical University General Hospital Ethical Committee (IRB2022-WZ-150). Human participants' names have been removed from all sections of the manuscript. Tianjin Medical University General Hospital Ethical Committee waived the need for informed consent. A total of 455 patients with AAAs underwent elective EVAR procedures between January 2018 and December 2021 at our single center. This study was performed in October 2022, and the authors were able to access the information of individual participants during or after data collection. Finally, 283 patients with infra-AAAs were enrolled in the analysis, excluding cases that did not meet the inclusion criteria. The patients were divided into two groups: those with T2EL-re-intervention (n = 42) and non-T2EL (n = 241). The exclusion criteria are described in a flowchart (Fig. 1). All patients were scheduled for follow-up at 1, 3, and 6 months using color Doppler ultrasound. If the occurrence of T2EL was found using color Doppler ultrasound, computed tomography angiography (CTA) inspection would be performed to confirm the diagnosis and evaluate whether re-intervention was required. CTA inspection was performed at 12 months and annually thereafter when the occurrence of T2EL was not identified by color Doppler ultrasound for monitoring. The patients were requested to the hospital immediately if they had any discomfort or recurrent symptoms. The endpoint of follow-up was the latest imaging or re-intervention due to T2EL.

### Preoperative morphological features

**Figure 2:**
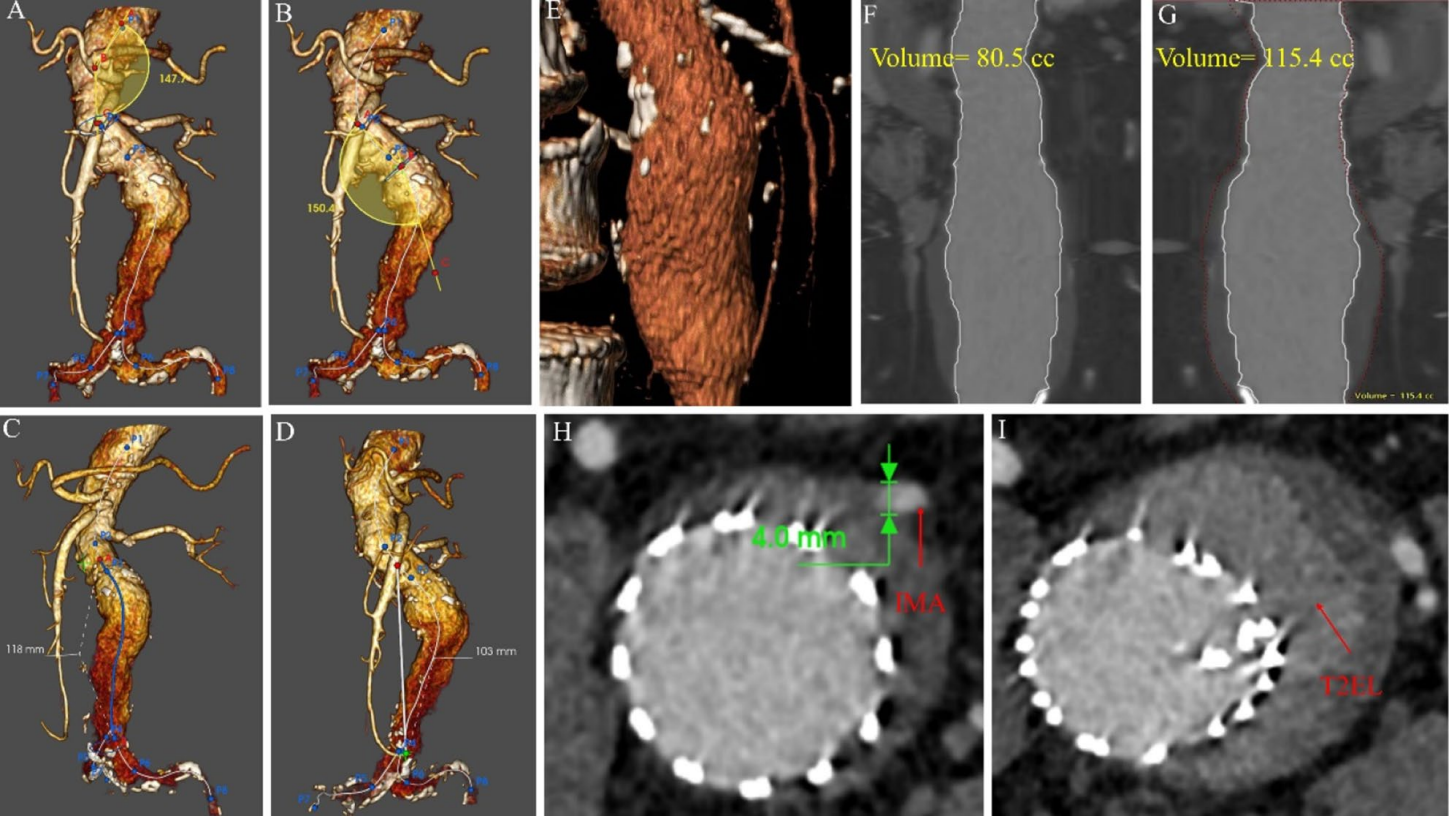
The morphological features of abdominal aortic aneurysm. (**A**) Angle (α) is defined as the angle between the central line of the upper abdominal aorta of the kidney and the central line of the lower abdominal aortic aneurysm neck. (**B**) Angle (β) is the angle between the aneurysm neck and the centerline of the aneurysm body. (**C**) L1 is defined as the length of the central line of the aneurysm. (**D**) L2 is defined as the length of the distance at the beginning to the end of the aneurysm. (**E**) The three-dimensional reconstruction of aneurysm sac by 3-Mensio software for estimating the proportion of thrombus. (**F**) The volume of blood in the aneurysm sac. (**G**) The volume of the whole aneurysm sac. (**H**) The diameter of the inferior mesenteric artery was measured by Endosize. (**I**) The occurrence of T2EL was identified by CTA.

All patients were screened according to the morphology of the aortic aneurysm on CTA. Morphological features were measured using Endosize (Therenva, France) and 3-Mensio vascular (Pie Medical Imaging, Netherlands) software. The measurement methods have been described previously^11,12^. Definitions of preoperative variables followed the reporting standards of the Society for Vascular Surgery (SVS) and the International Society for Cardiovascular Surgery (ISCS)^13,14^. The aneurysm features included aneurysm neck features; the neck angle (α), or the angle between the central line of the upper abdominal aorta of the kidney and the central line of the lower AAA of the neck (Fig. 2A); and the neck angle (β), or the angle between the aneurysm neck and the centerline of the aneurysm body (Fig. 2B). The tortuosity index of the aneurysm was defined as the length of the central line of the aneurysm divided by the length of the distance at the beginning and end of the aneurysm (Figs. 2C and 2D).

The aneurysm body volume (ABV) (Fig. 2F), aneurysm thrombus volume (ATV) (Fig. 2G), and the ABV/ATV rate (%VT) were calculated semi-automatically from the lowest renal artery and the aortic bifurcation using the dedicated vessel analysis software 3Mensio Vascular. The arterial diameter was measured using EndoSize software (Fig. 2H), and the occurrence of T2EL was identified by CTA (Fig. 2I).

### *Statistics *analysis

Descriptive analyses were performed to report the clinical characteristics and outcomes of the cohort. Values are presented as frequencies or percentages for categorical factors and were calculated using the chi-square test or Mann–Whitney U test. The mean ± standard deviation (SD) for continuous variables was analyzed using Student’s t-test.

Multivariable analyses were conducted using a binary logistic regression model to determine the prognostic factors for EVAR-related T2EL, expressed as odds ratios (ORs) and 95% confidence intervals (CIs). A nomogram was developed based on the results of the multivariate analyses. The model was then subjected to 500 bootstrap resamples for internal validation of the same cohort. A concordance index (C-index) was used to determine the predictive performance of the risk model. It ranges from 0.5 to 1.0 (0.50–0.70, low accuracy; 0.71–0.90, moderate accuracy; 0.91–1.0, high accuracy). Calibration of the model for EVAR-related T2EL was performed by comparing the predicted risk with the observed risk after bias correction, and the Hosmer–Lemeshow goodness of fit test was performed to evaluate the stability of the model, with *P *> 0.05 indicating an excellent agreement between model-predicted and observed values. Moreover, a receiver operating characteristic (ROC) curve was generated to evaluate the discrimination of the risk model using the area under the curve (AUC), and an AUC of ≥ 0.7 was considered to have a good predictive value. The probability values were two-tailed, and a 5% significance level was considered. Data were analyzed using R version 4.1.2 (https://www.r-project.org/).

## Results

### Patient characteristics

All patients' demographic characteristics and follow-up periods are summarized in Table 1. The age of the T2EL-related re-intervention group was 73.04 ± 8.35 years, older than that of the non-T2EL group, 68.17 ± 7.33 years (*P *< 0.001), and more patients were exposed to smoking and hypertension in the T2EL-related re-intervention group (*P *= 0.005). The median period between re-intervention and the initial operation in the T2EL group was 178 days (interquartile range [IQR], 12.3–35.0), and the latest imaging median follow-up time in the non-T2EL group was 355 days (IQR, 162–646). In the T2EL-related re-intervention group, most patients were treated using Medtronic stents, whereas most patients in the non-T2EL group received Gore stents, and the treatment was determined according to physician's decision or patients’ anatomical features.

**Table 1:** Demographic characteristics of patients.

	T2EL-related re-intervention (n = 42)	non-T2EL (n = 241)	t/χ^2^/Z	*P*
Age(years)	73.04 ± 8.35	68.17 ± 7.33	3.891	< 0.001
Sex(male)	85.70%	83.40%	0.14	0.708
Smoking	73.80%	50.20%	8.041	0.005
Hypertension	73.80%	46.00%	11.018	0.001
Hyperlipidemia	47.60%	52.70%	0.369	0.543
Diabetes mellitus	42.80%	47.30%	0.284	0.594
COPD	26.10%	36.90%	1.805	0.179
Chronic renal insufficiency	42.80%	28.20%	3.625	0.057
Anticoagulation	14.29%	10.37%	0.561	0.454
Follow up time(days)	178(84,446)	355(162,646)		
**Stent graft**				
Medtronic	47.62%	29.87%		
Gore	23.81%	31.53%		
Cook	19.05%	11.61%		
Domestic brands	9.52%	26.97%		

*COPD* Chronic obstructive pulmonary disease.

### Abdominal* aortic aneurysm (AAA) morphological features*

The morphological features of the aneurysm neck and body are shown in Table 2. In terms of the aneurysm neck morphology features, the average maximum diameters in the T2EL-related re-intervention group were 2.76 ± 0.35 cm, while they were 2.81 ± 0.26 cm in the non-T2EL group. The aneurysm neck lengths were 2.02 ± 0.19 cm and 2.07 ± 0.27 cm in the T2EL-related re-intervention group and the non-T2EL group, respectively. The neck angles (α) were 146.75 ± 8.62 and 145.58 ± 2.05, while the neck angles (β) were 153.41 ± 8.43 and 155.99 ± 11.19 in the T2EL-related re-intervention group and non-T2EL group, respectively.

**Table 2:** Abdominal aortic aneurysm (AAA) morphological features.

	T2EL-related re-intervention (n = 42)	non-T2EL (n = 241)	t/χ^2^/Z	*P*
**Aneurysm neck features**				
Neck maximum diameter (cm)	2.76 ± 0.35	2.81 ± 0.26	− 0.83	0.408
Aneurysm neck length (cm)	2.02 ± 0.19	2.07 ± 0.27	− 1.29	0.197
Neck angle(α°)	146.75 ± 8.62	145.58 ± 2.05	1.844	0.066
Neck angle(β°)	153.41 ± 8.43	155.99 ± 11.19	− 1.42	0.155
**Aneurysm body features**				
Body maximum diameter (cm)	5.80 ± 1.06	5.78 ± 0.61	0.206	0.837
Aneurysm body volume (ABV), CC	175.1 ± 82.20	175.03 ± 0.49	0.44	0.662
Aneurysm thrombus volume (ATV), CC	72.05 ± 16.05	87.70 ± 9.54	− 6.13	< 0.001
%VT	41.13 ± 9.21	50.10 ± 5.45	− 6.13	< 0.001
Tortuosity index (L1/L2)	1.26 ± 0.10	1.25 ± 0.11	2.666	0.461
**Common iliac artery aneurysm**				
None	48.84%	60.74%	1.793	0.181
Unilateral	37.21%	26.45%	2.349	0.125
Bilateral	9.30%	12.81%	0.368	0.544
**Branching vessel features**				
Number of patency lumber arteries	4(4,5)	3(2,4)	7.188	< 0.001
Patency of inferior mesenteric artery (IMA)	85.70%	76.30%	1.813	0.178
Diameter of IMA (mm)	3.01 ± 0.46	2.63 ± 0.42	4.679	< 0.001

%VT = ATV/ABV*100%.

The average maximum diameter of the aneurysm body was 5.80 ± 1.06 cm, and the aneurysm body volume was 175.1 ± 82.20 cc in the T2EL-related re-intervention group, while these values in the non-T2EL group were 5.78 ± 0.61 cm and 175.03 ± 0.49 cc, respectively. The T2EL-related re-intervention group had a lower aneurysm thrombus volume (72.05 ± 16.05 cc) than the non-T2EL group (87.70 ± 9.54 cc) (*P *< 0.001). The %VT values (aneurysm thrombus volume/aneurysm body volume × 100%) were 41.13 ± 9.21% in the T2EL-related re-intervention group and 50.10 ± 5.45% in the non-T2EL group (*P *< 0.001). The tortuosity index (L1/L2) was 1.26 ± 0.10 in the T2EL-related re-intervention group, while it was 1.25 ± 0.11 in the non-T2EL group.

In the T2EL-related re-intervention group, 48.84% of the patients had no iliac aneurysm, 37.21% had a unilateral aneurysm, and 9.30% had a bilateral iliac aneurysm.

The median numbers of patent lumbar arteries were 4 (IQR, 4–5) in the T2EL-related re-intervention group and 3 (IQR, 2–4) in the non-T2EL group (*P *< 0.001). The average diameters of the inferior mesenteric artery were 3.01 ± 0.46 mm in the T2EL-related re-intervention group and 2.63 ± 0.42 mm in the non-T2EL group (*P *< 0.001).

### *Receiver *operating* characteristic (ROC) curve*

**Figure 3:**
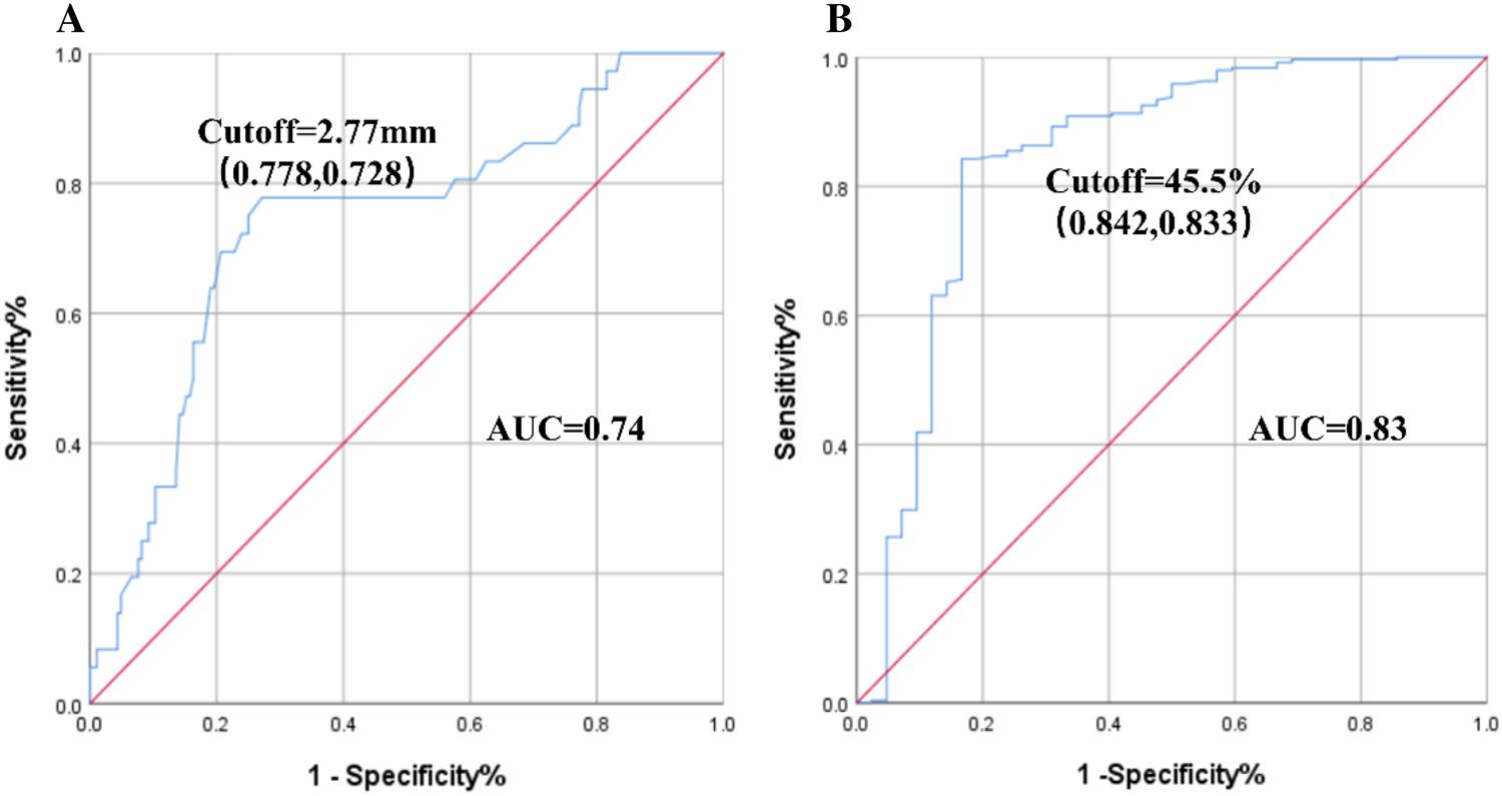
Receiver operating characteristic (ROC) curve identified the threshold of IMA and %VT. (**A**) The ROC curve identified the cutoff of the IMA diameter as 2.77 mm. (**B**) The ROC curve identified the cutoff of the thrombus proportion in the aneurysm sac as 45.5%.

Univariate analysis showed that the %VT of AAAs and the diameter of the inferior mesenteric artery may be related to re-intervention in patients with T2EL after EVAR. Thus, we constructed an ROC curve to determine the critical value that may lead to re-intervention. The ROC curve presented that the cutoff of the IMA diameter was 2.77 mm (sensitivity and specificity were 0.778 and 0.728, respectively), and the accuracy (AUC) was 0.74 (Fig. 3A). The cutoff of %VT was 45.5% (sensitivity and specificity were 0.842 and 0.833, respectively), and the accuracy (AUC) was 0.83 (Fig. 3B). The results indicate that patients with an IMA of > 2.77 mm and a %VT of < 45.5% have a higher risk of intervention due to T2EL after EVAR.

### *Variable *selection* and model development*

Seven clinical variables were screened as prognostic factors to predict re-intervention due to T2EL after EVAR using univariate analysis, including age, smoking, hypertension, %VT, number of patent lumbar arteries, and aneurysm thrombus volume, which were associated with a higher risk of re-intervention. According to multivariate analysis, except for %VT, hypertension, and aneurysm thrombus volume, age (OR, 1.172; 95% CI, 1.051–1.307; *P *= 0.004), smoking (OR, 13.418; 95% CI, 2.362–76.215; *P *= 0.003), diameter of inferior mesenteric artery (IMA) (OR, 21.380; 95% CI, 3.060–149.390; *P *= 0.002), and number of patent lumbar arteries (OR, 9.736; 95% CI, 3.175–29.857; *P *< 0.001) were associated with a higher risk of T2EL-related re-intervention (Table 3).

**Table 3:** Multivariate analysis on the risk factors that may lead to T2EL re-intervention.

	T2EL-related re-intervention (n = 42)	non-T2EL (n = 241)	t/χ^2^/Z	P*	Odds ratio(95%CI)	*P*^#^
Age(years)	73.04 ± 8.35	68.17 ± 7.33	3.891	< 0.001	1.172(1.051,1.307)	0.004
Smoking	73.80%	50.20%	8.041	0.005	13.418(2.362,76.215)	0.003
Hypertension	88.10%	72.20%	4.764	0.001	3.620(0.799,16.409)	0.095
%VT	41.13 ± 9.21	50.10 ± 5.45	− 6.126	< 0.001	0.144(0.019,1.092)	0.061
Number of patency lumber arteries	4(4,5)	3(2,4)	7.188	< 0.001	9.736(3.175,29.857)	< 0.001
Aneurysm thrombus volume(ATV), cc	72.05 ± 16.05	87.70 ± 9.54	− 6.133	< 0.001	2.426(0.779,7.555)	0.126
Diameter of IMA (mm)	3.01 ± 0.46	2.63 ± 0.42	4.679	< 0.001	21.380(3.060,149.390)	0.002

P*, t/χ^2^/Z; odds ratio and *P*^#^, logistic regression analysis.

### Nomogram and validation

**Figure 4:**
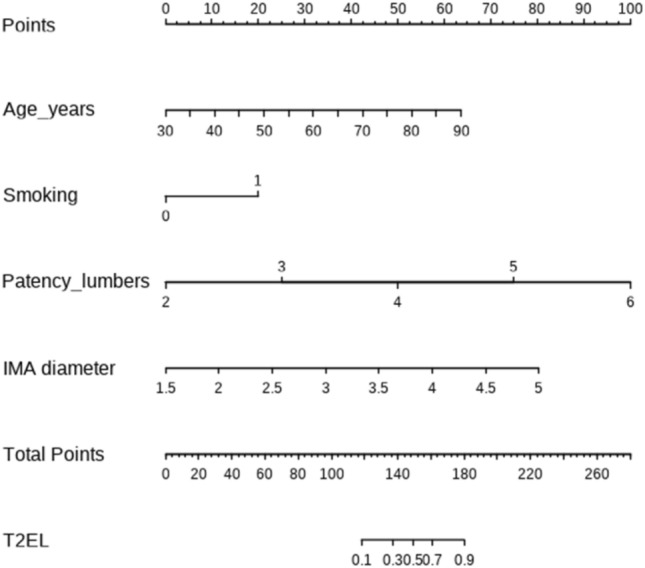
A nomogram for risk factors. Each of the four risk factors was assigned a score on the points scale. After adding each score together and locating the value on the total points scale, a straight line could be drawn downward to determine the estimated risk of adverse events.

**Figure 5:**
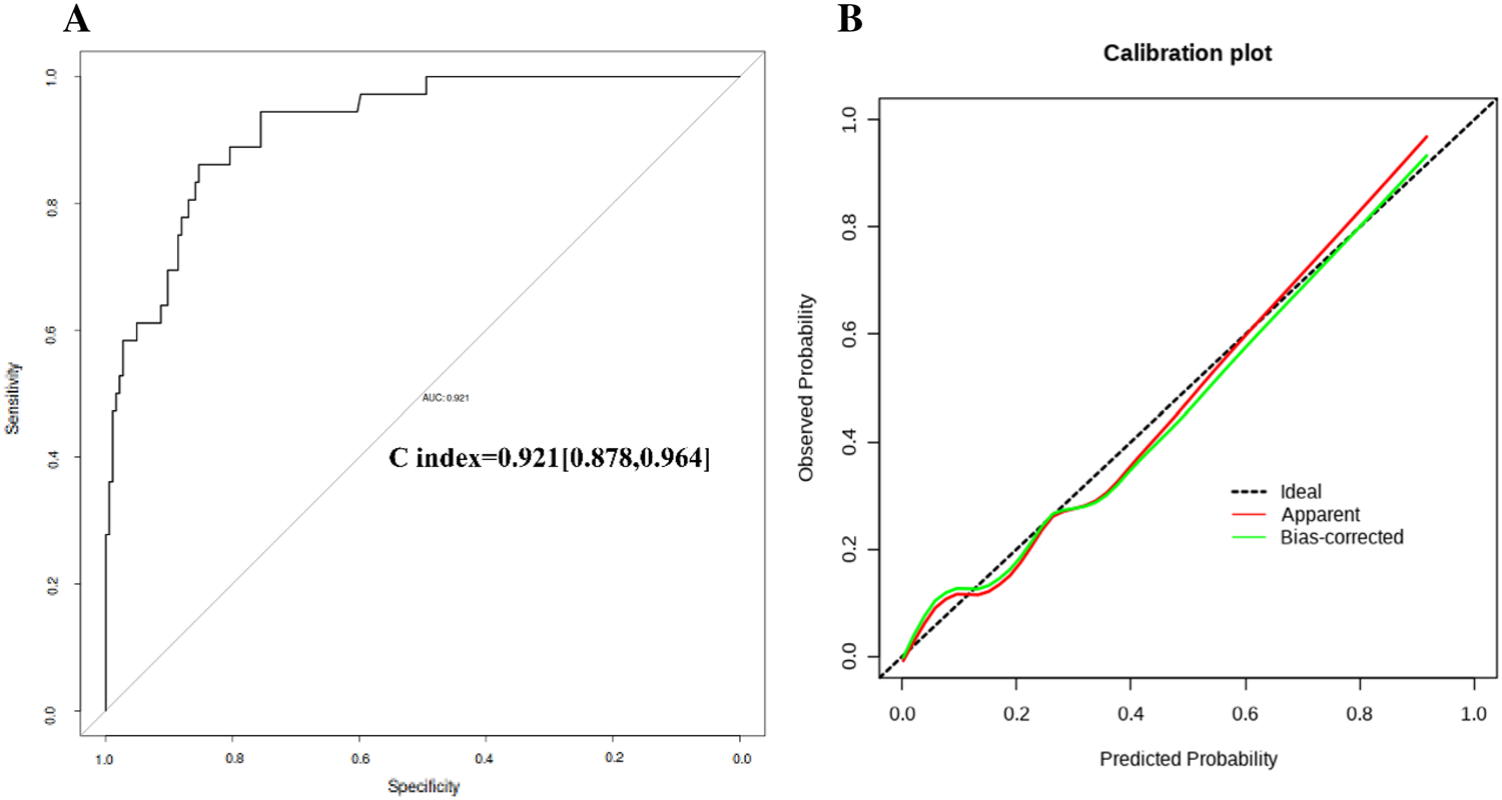
The C-index and calibration plots for validating the nomogram risk model. (**A**) The C-index value was 0.921(sensitivity and specificity were 0.878 and 0.964, respectively), which indicated a moderate accuracy for the nomogram risk model. (**B**) The calibration plots for the nomogram risk model present an excellent agreement between model prediction and observed risk.

A nomogram integrating all the significant prognostic factors was established (Fig. 4). The nomogram illustrated that the number of patent lumbar arteries was the largest contributor to poor prognosis, followed by age, smoking, and diameter of inferior mesenteric artery. Each of the four risk factors was assigned a score on a point scale. After adding each score together and locating it on the total point scale, a straight line was drawn downward to determine the estimated risk of adverse events. The C-index for this established risk model to predict T2EL-related re-intervention after EVAR was 0.921 (sensitivity and specificity were 0.878 and 0.964, respectively), which indicated a high accuracy (Fig. 5A). The Hosmer–Lemeshow goodness of fit test was performed on the model, and the chi-square value was 3.210 (*P *= 0.920). The calibration plots presented an excellent agreement between the model-predicted and observed risks of re-intervention within the same group (Fig. 5B).

## Discussion

We built and verified a nomogram model for individually predicting patients with T2EL (exposed to age, smoking, larger diameter of inferior mesenteric artery, and number of lumbar arteries) who are more likely to encounter re-intervention. We also determined that an IMA diameter of > 2.77 mm and a < 45.5% proportion of thrombus volume may be risk factors for re-intervention. The nomogram incorporated demographic information, medical history, and aneurysm anatomical characteristics in patients with AAA and showed good discrimination and calibration performance. Thus, it can provide effective assistance for preventing re-intervention in patients with T2EL. Meanwhile, the ROC curve analysis resulted in two novel thresholds which may cause patients with T2EL to consider re-intervention, despite the thresholds exhibiting acceptable differences compared with previously reported values, such as an IMA diameter of 2.5 mm^15^ or 3.5 mm^16^ and a thrombus volume of < 40.0%^11^. For AAA patients, pre-emptive embolization of the risky aneurysm sac side branches can effectively and safely reduce the incidence of T2EL^17^ and T2EL-related re-intervention^18^ after EVAR. For a patient with AAA diagnosed in the clinic, it is possible to quickly and easily estimate the possibility of re-intervention once T2EL has occurred using the novel nomogram model and thresholds, effectively working as a measure to avoid re-hospitalization and additional financial burdens for these patients, as well as to alleviate the financial deficit of the medical insurance system. Thus, the application of this prediction model to prevent re-intervention of patients with AAAs has both patient and economic benefits.

In a previous study, several risk factors were determined that may increase the incidence of T2EL after EVAR, such as older age, chronic renal failure, chronic obstructive pulmonary disease (COPD), smoking, hypertension, and other anatomical features of the aneurysm^19,20^. In our predictive model, older age and smoking are also independent risk factors that may increase the incidence of re-intervention in patients with T2EL. In the recent largest prospective study in Japan^21^, the researchers examined the medical records of 17,099 patients who underwent EVAR for AAA, and the results showed that age is an independent risk factor for T2EL, which indicates that advanced age has a more important influence on the occurrence of T2EL, in agreement with other previously published studies^7,21^ and our present research. Smoking is considered important risk factor for the occurrence and development of AAA, and smoking cessation can effectively reduce the AAA rupture rate by 20%^22^. Meanwhile, smoking can also prompt the occurrence of T2EL, as identified by the aforementioned largest prospective study^21^, recent meta-analyses^10,23^, and other retrospective studies^16,24^. Thus, patients with T2EL-related risk factor should be considered when implementing pre-embolism treatment during primary EVAR intervention.

Surgeons often have more interest in the relationship between the feeding arteries of the aneurysm sac, anatomical features of the aneurysm body and neck, thrombus proportion of the aneurysm, and T2EL-related re-intervention. These factors can be treated by endovascular surgery. T2ELs are formed by incompletely occluded branch arteries that continuously provide blood to the gap between the stent graft and the arterial wall; thus, T2EL generally originates from the inferior mesenteric artery, lumbar artery, and accessory renal artery, which are the main branches of an infrarenal AAA. The inferior mesenteric artery has the largest diameter of the main branches from the perspective of previous studies, and an inferior mesenteric artery diameter of > 2.5 mm^15,25^ or > 3.5 mm^16^ may increase the incidence of T2EL after EVAR. In addition, an increasing number of patent lumbar arteries may also increase the occurrence of T2EL after EVAR. In a recent study, 5.5 patent lumbar arteries were defined as the threshold that may promote the incidence of T2EL after EVAR^16^. It is widely accepted that the maximum diameter and number of feeding arteries are significant risk factors for T2EL. In our study, we also identified that the diameter and number of feeding arteries are independent risk factors for T2EL-related re-intervention. The T2EL-re-intervention group had a larger diameter of the inferior mesenteric artery, and through ROC curve analysis, the threshold was defined as 2.77 mm. Logically, a larger diameter of the feeding artery yields more difficult occlusion, which can lead to persistent feeding to the gap between the stent graft and the aorta wall, promoting the formation of T2EL. ROC curve analysis showed good sensitivity and specificity. Thus, we have reason to believe that feeding artery diameter is a risk factor for T2EL-related re-intervention, as the diameter of the inferior mesenteric artery was > 2.77 mm. The number of patent lumbar arteries is another risk factor for re-intervention. Recent studies^16,24,26^ stated that a higher number of patent lumbar arteries, ranging from four to six, may increase the incidence of T2EL. In our study, the results of univariate and multivariate analyses showed a significant statistical difference between the four patent lumbar arteries in the T2EL-related re-intervention group and three patent lumbar arteries in the non-T2EL group, indicating that if patients have a higher number of patent lumbar arteries, they would be more likely to suffer a re-intervention after EVAR. Thus, pre-embolism treatment may be beneficial in these patients. Interestingly, thrombus in the aneurysm sac may be a protective factor against T2EL-related re-intervention. According to a previous study, patients with < 40% thrombus in the aneurysm are more likely to require re-intervention after EVAR^11^. However, in previous research on the mechanism of AAAs, intraluminal thrombus was defined as a negative role that may aggravate tissue oxidative stress reaction and promote the recruitment of inflammatory factors and lead to the development of AAAs. Thus, it is difficult to define the exact role of intraluminal thrombi in AAAs. In the present study, patients from the T2EL group had a lower proportion of intraluminal thrombus than those from the non-T2EL group, and the ROC curve analysis showed a threshold of 45.5%, which manifested a positive role to prevent re-intervention in the non-T2EL group. The existence of an intraluminal thrombus may assist in occlusion of the branches and decrease the residual flow between the stent graft and artery wall, thus decreasing the need for T2EL-related re-intervention. In summary, using this prediction model, we proposed that patients with a high prediction probability of re-intervention should undergo pre-embolism treatment in their primary EVAR treatment.

### Limitations

The current study has some limitations. First, this was a small-sample, retrospective, single-center study. It is necessary to conduct prospective research in a large multicenter study to confirm the practicality of the nomogram. Furthermore, due to the limited number of patients, we did not distinguish between early and late T2EL or consider the influence of risk factors on the different stages of T2EL, which could be studied in a larger cohort in the future. Finally, we did not analyze the relationship between the application of antiplatelet drugs and hemodynamic features and the possibility of T2EL-related re-intervention, and we did not stratify the patients based on the application of anticoagulants, which should be explored in the future.

## Conclusion

In conclusion, EVAR is a feasible and safe treatment option for most patients with AAAs. We developed and validated a novel risk model for predicting the risk of T2EL-related re-intervention in these patients. Through this model, operators could more precisely estimate the risk of individual patients after EVAR and identify subgroups of patients who may require pre-embolism treatment in the first operation and more intensive imaging supervision.

## Data Availability

The datasets used and/or analyzed during the current study are available from the corresponding author on reasonable request.

## Abbreviations

AAA: Abdominal aortic aneurysm
EVAR: Endovascular aneurysm repair
T2EL: Type II endoleak
ROC curve: Receiver operating characteristic curve
